# Reconstruction of hip flexion after arthroscopic iliopsoas tendon release using free functional muscle

**DOI:** 10.1016/j.jpra.2025.08.003

**Published:** 2025-08-08

**Authors:** B. Schäfer, J. Bahm, J.P. Beier

**Affiliations:** Department for Plastic Surgery, Hand Surgery—Burn Center, Division for Plexus Surgery, University Hospital RWTH Aachen, Pauwelsstraße 30, Aachen, 52074 North Rhine-Westphalia, Germany

**Keywords:** Free functional muscle transfer, Hip flexion reconstruction, Iliopsoas muscle replacement, Functional reconstruction, Lower limb reconstruction, Arthroscopic iliopsoas release

## Abstract

We present the case of a physically active 50-year-old patient who underwent an arthroscopic release of the iliopsoas tendon due to a snapping hip. Postoperatively, active hip flexion was severely weakened. As a consequence patient had no stable gait pattern and was unable to continue her sports and physical activities. An MRI scan revealed an atrophy of the iliopsoas muscle. We reconstructed the patient's hip flexion function using a free functional muscle transfer of the ipsilateral gracilis muscle. After reinnervation of the microsurgically transferred gracilis muscle, the patient developed a strong hip flexion again, so that her gait pattern returned to normal and she was able to do strength training and ride a bicycle again.

This case shows a very rare but serious complication of the frequently performed iliopsoas release. Functional reconstructions of hip flexion have only been described in single cases. To our knowledge, no case of free functional muscle transfer for the reconstruction of the hip flexion has been described in the literature. We can present a successful complex method for active reconstruction.

## Introduction

Arthroscopic tendon release or tenotomy of iliopsoas muscle is a common treatment for painful snapping hips.^1^ However, it is controversial discussed.^2^ It can lead to hip flexion weakening, hip instability and iliopsoas muscle atrophy, if active motion turns to nearly zero following complete tenotomy. Gouveia et al.^2^ showed in a review published in 2021 that the procedure is performed mostly in young, female patients (28 years, 82 % female). They reported a sufficient treatment of hip snapping in 93 % of cases and an improvement in function in all summarized studies. A minority of included studies (6 studies) investigated postoperative hip flexion strength. Reduction in strength was observed in one-third-of the studies.^2^ No weakening was detected in other studies.^3^ Radiologically, atrophy of the iliopsoas muscle was observed in about 92 % of cases. Overall, there were no serious complications and a return to sport and activity was possible despite initial reductions in strength.

However, a complete paralysis or lesion of the iliopsoas muscle ultimately leads to weakness or inability to bend the hip. One case report presents a patient with luxation of the hip following arthroscopy, where tenotomy of the iliopsoas tendon may have had an influence.^4^

### Case presentation

A 50-year-old female patient in an athletic general condition (BMI: 19.5 kg/m²) presented in our outpatient department. She suffered from pain in the right hip area for approx. 6 months so that an arthroscopy of the hip joint was indicated elsewhere. As part of hip arthroscopy 11 months before first presentation, according to the operation records the iliopsoas tendon was “released.” Subsequently, a previously non-existent paresis of the hip flexion occurred. Initially there was a suspicion of nerve damage, i.e. to the lumbosacral plexus / the femoral nerve. A neurological assessment was performed, but a nerve lesion could be excluded by neurography.

**Figure 1:**
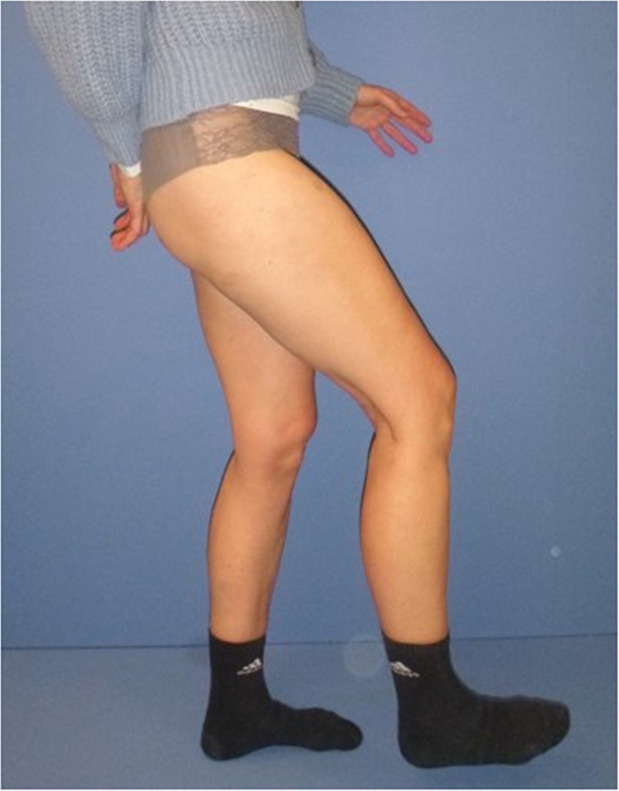
Maximum hip flexion at the first presentation in our clinic, 11 months after arthroscopic release of the ilipsoas tendon.

**Figure 2:**
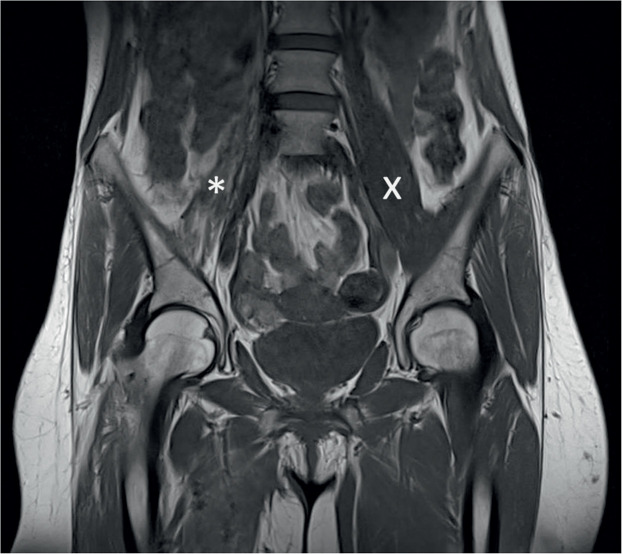
Atrophic psoas muscle (*) compared to the uninjured contralateral muscle (x) in a MRI scan 6 months after tendon release.

At the time of examination, the patient was dependent on crutches for longer walking distances and was significantly restricted in everyday life (Video 1). Clinically, the examination revealed a degree of strength of 2/5 for right-sided hip flexion (Figure 1). An MRI scan showed severe atrophy of the right iliopsoas muscle with remodeling (Figure 2).

### Planning and surgery

We aimed to reconstruct the hip flexion by replacing the damaged muscle using a free functional muscle transfer (FFMT). The femoral nerve’s function was regular, to be used as donor nerve. Patency of the inferior epigastric vessels as recipient vessels for the gracilis vascular pedicle was confirmed by CT-angiography. The ipsilateral gracilis muscle was planned as donor.

**Figure 3:**
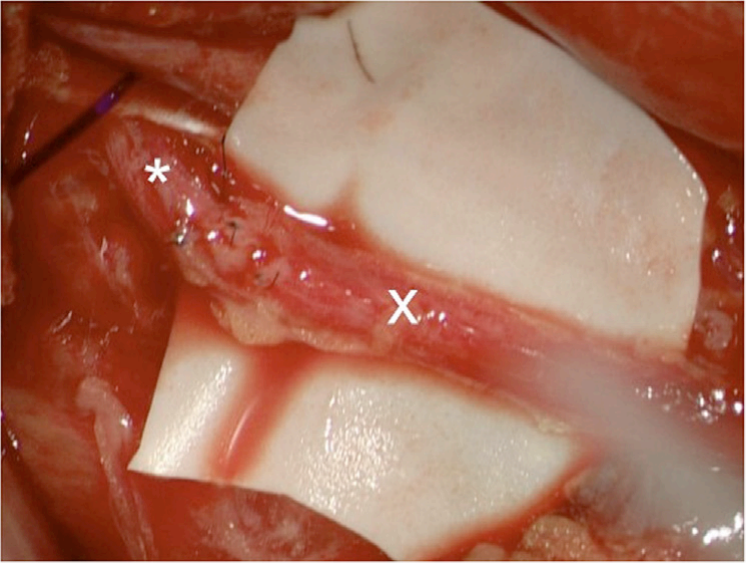
Nerve coaptation of a fascicle of the femoral nerve (*) and the obturator nerve (x).

The procedure was performed in the supine position. A subcostal approach was used. The inferior epigastric artery and vein were prepared for microsurgical anastomosis. In addition, the femoral nerve was visualized and stimulated. Two redundant fascicles were identified to serve as motor nerve donor. Supplementary an intraoperative neuropathological frozen section showed regular nerve quality. In addition, the now inactive distal insertion of the atrophic iliopsoas muscle was dissected via an approach below the inguinal ligament, serving as tendinous insertion for later gracilis muscle fixation. After raising the gracilis muscle, including its vascular pedicle and its motor branch, it was firstly inserted retroperitoneally and fixed in the area of the lumbar vertebrae as new origin and, after tunneling along the original course of the muscle, distally at the insertion of the iliopsoas muscle in a flexed position of the hip of 70° Microsurgical end-to-end anastomosis of the arteries (8–0 interrupted suture) and the veins (2.5 mm ring-pin coupler anastomosis) using the FLOW COUPLER Device® (Synovis Micro Companies Alliance,Inc.,Birmingham,AL 35211,USA) for post-operative monitoring of the completely buried flap was performed. Finally, the femoral nerve fascicle and the gracilis branch from the obturator nerve were coaptated end-to-end using 9–0 interrupted sutures (Figure 3). The length of the obturator branch was approx. 9 cm, so that at least 3 months were expected for nerve growth until the transferred muscle could be reached. From this point in time axons begin to grow into the muscle and a gradual increase in function, albeit over a period of months, can be expected.^5^ The patient was immobilized postoperatively in an individually adapted orthosis with approx. 70° flexion in the hip for 6 weeks.

### Follow up

**Figure 4:**
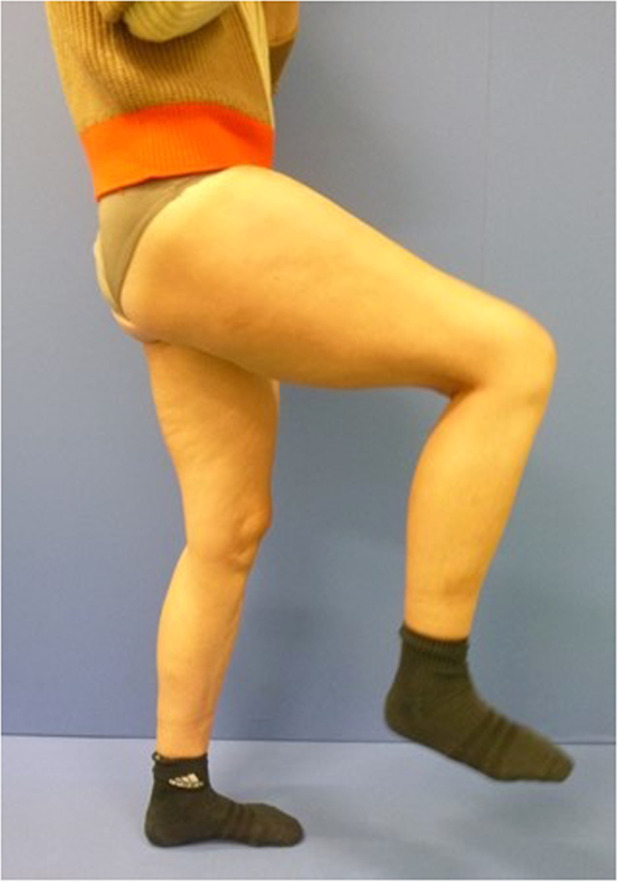
Stabilized hip flexion one year after FFMT.

After the individual splint was removed, the hip was gradually mobilized with physiotherapeutic support. Further course was uneventful. The patient underwent intensive rehabilitation. Active and passive movement exercises were carried performed. Electrical stimulation during the denervated phase of the muscle after the transfer was not possible due to the anatomical position of the graft in the retroperitoneal space. 8 months postoperatively, the patient was already able to achieve a hip flexion of up to approx. 70° standing upright (Video 2). 12 months postoperatively, hip flexion of at least 80° was possible in the standing position (Figure 4) and up to approx. 115° in the lying position. The patient reported that she was able to do regular weight training and ride her bicycle. The gait had clearly normalized and crutches were no longer required (Video 3). No restriction due to the ipsilateral removal of the gracilis muscle could be determined.

## Discussion

Arthroscopic release of the iliopsoas tendon is a commonly used method in snapping hips. The reduction in hip flexion is described as being initial problem which can lead to crutch use for up to 8 weeks,^6^ but is completely normalized after 12 months.^1^ However, studies show that on average there is a long-term reduction in hip flexion strength of 19 % with a loss of muscle volume of 25 %.^7^ There are no reports in the literature of a pronounced weakening of hip flexion with significant impairment of gait and everyday life.

This patient had a pronounced weakness with significant restrictions on her everyday life and sportive activity. We had to find a way to restore active hip flexion and the necessary strength for a stable gait. To the best of our knowledge, no method for restoring active hip flexion including original arbitrary control of this movement has been described so far. Case reports present individual options for restoring hip flexion. In one of the few reported cases, Marsden and Grinsell described a sarcoma patient with resection of the iliopsoas muscle. They performed a pedicled transfer of the rectus abdominis muscle for successful reconstruction of hip flexion.^8^ However, in this case regained hip flexion did not result from actively controlled femoral nerve branch activation. In our case, we were able to demonstrate the first-time use of a FFMT on the femoral nerve as a successful treatment option for this condition achieving an orthotopic dynamic restoration of hip flexion.

However, the risk of possible free flap loss and the unpredictability of nerve reinnervation associated with a FFMT should be critically noted in our procedure. Due to the buried muscle the postoperative flap perfusion monitoring was only possible using the FLOW COUPLER Device®. It should also be mentioned that the operation takes around 9 h, which is necessary for a procedure of this scope, as only small parts of the operation can be performed simultaneously. In addition, microsurgical expertise and equipment is required. The donor-site morbidity of the gracilis muscle is low, especially in comparison to the transfer of the rectus abdominis muscle.^9^, ^10^, ^11^ Our patient had no complications at the donor-site.

## Conclusion

We can show that successful treatment of the rare entity of hip flexion weakness or complete loss of hip flexion, which severely restricts everyday life, is possible with a FFMT. The presented case shows that it is even possible to regain a normalized gait and to resume sporting activities such as weight training and cycling.

### Statements

The patient has given her full consent to the publication of her data, photos and videos. A positive ethics vote for the corresponding evaluation of the data by the local ethics committee is available (EK number 077/21).

Video 1: The patient's preoperative gait pattern after the arthroscopic ilipsoas release.

Video 2: Hip movement 8 months after FFMT.

Video 3: Normalized gait pattern 1 year after FFMT. The patient is active in sports again and has no restrictions in everyday life.

## Declaration of competing interest

The authors declare that they have no known competing financial interests or personal relationships that could have appeared to influence the work reported in this paper.

## Appendix Supplementary materials

Image, video 1

Image, video 2

Image, video 3
